# Multisystem inflammatory syndrome in children characterized by enhanced antigen-specific T-cell expression of cytokines and its reversal following recovery

**DOI:** 10.3389/fped.2023.1235342

**Published:** 2023-12-05

**Authors:** Nathella Pavan Kumar, Kadar M. Abbas, Rachel M. Renji, Aishwarya Venkataraman, Arul Nancy, Poovazhagi Varadarjan, Elilarasi Selladurai, Thankgavelu Sangaralingam, Ramya Selvam, Akshith Thimmaiah, Suresh Natarajan, Ganesh Ramasamy, Syed Hissar, Uma Devi Ranganathan, Thomas B. Nutman, Subash Babu

**Affiliations:** ^1^ Department of Immunology, ICMR—National Institute for Research in Tuberculosis, Chennai, India; ^2^ National Institutes of Health—International Center for Excellence in Research, Chennai, India; ^3^ Department of Clinical Research, ICMR—National Institute for Research in Tuberculosis, Chennai, India; ^4^ Department of Pulmonology, Institute of Child Health and Hospital for Children, Chennai, India; ^5^ General Pediatrics, Dr. Mehta’s Children’s Hospital, Chennai, India; ^6^ General Pediatrics, Rainbow Children’s Hospital, Chennai, India; ^7^ Laboratory of Parasitic Diseases, National Institute of Allergy and Infectious Diseases, National Institutes of Health, Bethesda, MD, United States

**Keywords:** MIS-C, SARS-CoV-2, COVID-19, CD4^+^ T cells, CD8^+^ T cells, cellular immune responses

## Abstract

**Background:**

Multisystem inflammatory syndrome (MIS) in children is considered to be a post-infectious complication of COVID-19. T-cell responses in children with this condition have not been well-studied.

**Methods:**

We aimed to study the immune responses in children with MIS in comparison to children with acute COVID-19 and children with other infections. Whole blood was stimulated with severe acute respiratory syndrome coronavirus-2 (SARS-CoV-2)–specific antigens and flow cytometry was performed to examine CD4^+^ and CD8^+^ T-cell responses.

**Results:**

Children with MIS had higher frequencies of CD4^+^ and CD8^+^ T cells expressing cytokines at baseline and upon SARS-CoV-2 antigen–specific stimulation in comparison to children with COVID-19 and/or other infections. Children with COVID-19 also exhibited higher frequencies of CD4^+^ and CD8^+^ T cells expressing cytokines at baseline and upon SARS-CoV-2 antigen–specific stimulation in comparison to children with other infections. At 6–9 months following treatment and recovery, this enhanced response against SARS-CoV-2 antigens was down modulated in children with MIS.

**Conclusion:**

Our study, therefore, provides evidence of enhanced activation of CD4^+^ and CD8^+^ T-cell responses in children with MIS and reversal following recovery.

## Introduction

Severe acute respiratory syndrome coronavirus-2 (SARS-CoV-2) caused the pandemic that surfaced at the end of 2019, known globally as COVID-19. The disease resulted in a high rate of morbidity and mortality across the globe. Although, the infection rate in children was similar to that of adults with respect to viral load (1), they were found largely to have less severe disease, perhaps as a result of better immune function or differential expression of the angiotensin-converting enzyme 2 (ACE2) receptors (2). Multisystem inflammatory syndrome (MIS) is a life-threatening inflammatory condition that affects the heart, lungs, kidneys, brain, skin, eyes, and gastrointestinal organs of children (MIS-C) and adolescents (MIS-A) (3). Their clinical presentations are similar to that of patients with Kawasaki disease (KD), toxic shock syndrome (TSS), and secondary hemophagocytic lymphohistiocytosis (SHLH) (4, 5). The manifestations of MIS-C were reported in pediatric cases in COVID-19 hotspots, and the Centers for Disease Control and Prevention (CDC) reported this condition in April 2020 (3).

The causes of MIS-C are unclear, but it is thought to be due to immune dysregulation. Initially, it was hypothesized that MIS-C was related to post-COVID-19 immune dysregulation based on laboratory reports (6). A major fraction of the children diagnosed with MIS has been reported to be seropositive for SARS-CoV-2-specific antibodies. However, most cases remain negative for viral RNA in RT-PCR (7, 8). The presence of autoantibodies with multiple antigenic targets has also been reported in acute MIS-C (9), though the role of autoantibodies has not been shown to be causal as of yet (10). Moreover, a genetic component to the occurrence of MIS-C has also been recently described (11, 12).

A few studies have focused on identifying the immune parameters at the cellular level that might help explain the differences between MIS-C and those from other groups (8, 10). Studies of components of the innate immune response in MIS-C showed marked neutrophil activation, degranulation signatures, and neutrophil extracellular traps (13). Rajamanickam et al. explored cellular biomarkers for MIS-C, inclusive of B cells, dendritic cells, monocytes, and T cells, which significantly distinguish MIS-C from other diseases that have overlapping clinical symptoms (14). Moreover, HLA-genotype alterations in the T cells of children with MIS were reported as MIS-C biomarkers (15). Similarly, there are studies demonstrating that following T-cell stimulation by SARS-CoV-2-specific antigens in children with MIS, there was a dysregulated increase of Type 1, Type 2, Type 17, and other pro-inflammatory cytokines and chemokines when compared to the healthy controls (16). It was also reported that T cells with specific β-chain receptors are minimally responsive to SARS-CoV-2-specific antigens but do respond to non-viral antigens in the majority of the children with MIS (17, 18). Although reported as a post-infectious complication, MIS-C has been found to have its own distinct features when compared to the acute SARS-CoV-2 infection. In most of the previously published studies, the comparison was either between the pediatric or adult COVID-19 groups or the healthy controls. In this study, we have compared children with MIS with other infections that had similar clinical presentations as MIS-C. Our study focused on identifying differences in the immunological responses to SARS-CoV-2 antigen stimulation at a cellular level in three patient groups—MIS-C, acute COVID-19, and other infections.

## Materials and methods

### Ethics statement

Informed consent was obtained from parents/guardians of all children along with assent where appropriate. The Internal Ethics Committee (IEC) of the participating institutes approved the study.

### Study population

Children, both male and female, between the ages of 12 months and 15 years, with clinical presentations of MIS-C, acute COVID-19, other infectious diseases [dengue fever, scrub typhus fever, and *Salmonella typhi* infection (enteric fever)] admitted to the Institute of Child Health, Dr. Mehta's Children Hospital, and Rainbow Children's Hospital, between December 2020 and May 2021, were included in this study. The study population was classified into three groups: MIS-C (*n* = 15), acute COVID-19 (*n* = 10), and other infectious diseases (*n* = 10). In the MIS-C group, eight children were evaluated 6–9 months following release from the hospital. The demographic and epidemiological data for this cohort have been previously reported (16) (Table 1).

**Table 1:** Demographics and hematology parameters of the study population.

	MIS-C		Acute COVID-19	Other infectious diseases
	During MIS-C	After MIS-C		
	*n* = 15	*n* = 8	*n* = 10	*n* = 10
Age median (years)	4.5 (1–13)	9 (3–12)	2 (1–13)	3 (1–10)
Male, *n* (%)	9 (60%)	7 (63.6%)	5 (50%)	3 (30%)
RT-PCR positive, *n* (%)	2 (1.3%)	NA	7 (70%)	0
Serology IgG positive, *n* (%)	15 (100%)	4 (36.36%)	1 (10%)	3 (30%)
CRP (<3 mg/L)	5 (50%)	0 (0%)	7 (46.6%)	2 (20%)
WBC 10^3^ cells/μl geometric mean/range	8.16 (3.65–13.29)	7.27 (5.35–9.51)	5.71 (4.41–8.19)	11.28 (5.13–29.83)
Hb (g/dl), geometric mean/range	10.15 (6–16.1)	12.17 (10.94–13.14)	10.68 (8.3–13.09)	10.9 (7.39–14.33)
Lymphocyte (%), median (range)	39.53 (4.85–63.95)	44.97 (36.3–62.99)	71.05 (12.45–80.4)	57.65 (14.13–63.22)
Neutrophils (%), median (range)	76.50 (32–89)	39.76 (23.28–53.3)	26.55 (10.59–82.14)	31.13 (22.97–83.25)
Platelets (200–450) × 10^9^/L, median (range)	267.3 (84.8–355.5)	295.54 (151.6–433)	264.55 (174.8–367.9)	339.3 (28.7–1,044.6)
Sodium (135–145 mmol/L), median (range)	132.5 (124–137)	—	NA	NA
Ferritin (ng/ml), median (range)	762.5 (306.1–5,377)	—	NA	NA
Median duration of stay (days)	7.5 (2–13)	NA	4 (2–6)	6.5 (3–7)
Dengue serology or NS1 positivity	—	—	—	4
Scrub typhus (IgM)	—	—	—	4
Enteric fever (culture confirmed)	—	—	—	2

### Sample collection

Blood was collected in heparin tubes (BD Biosciences) and processed within 4 h of collection. Sampling at baseline in all children was done prior to them being given any form of immunomodulatory treatment. Study staff involved in immunological assays was blinded to all clinical data, and all the collected samples from the different collaborating hospitals were processed at the NIH-ICER Lab. Acute COVID-19 disease and severity of COVID-19 were defined according to the Ministry of Health and Family Welfare (MoHFW) guidelines. Diagnosis and treatment of MIS-C was done according to the CDC definition for MIS-C. Dengue fever was confirmed by either serology or NS1 positivity. Scrub typhus was confirmed by serology. Confirmed culture was used to diagnose enteric fever.

### *In vitro* culture

Whole-blood cell cultures were performed to determine the intracellular levels of cytokines. Briefly, whole blood was diluted 1:1 with an RPMI-1640 medium, supplemented with penicillin/streptomycin (100 U/100 mg/ml), L-glutamine (2 mM), and (4-(2-hydroxyethyl)-1-piperazineethanesulfonic acid) (10 mM) (all from Invitrogen, Carlsbad, CA, USA), and distributed in 12-well tissue culture plates (Costar). The cultures were then stimulated with SARS-CoV-2 S-RBD (Receptor Binding Domain), SARS-CoV-2 ICL (Irradiated Cell Lysate), or *Mycobacterium tuberculosis M.tb* WCL (Whole Cell Lysate, H37Rv, BEI Resources) (non-viral antigen control) or media alone in the presence of the co-stimulatory molecules (CD49d/CD28) at 37° for 12 h. Brefeldin A (10 μg/ml) was added 4 h prior to harvest. After 12 h, centrifugation, red blood cell lysis, and washing were performed. The cells were then washed and permeabilized with BD Perm/Wash buffer (BD Biosciences) and stained with intracellular markers for an additional 60 min before washing and acquisition. Antibodies containing surface and intracellular markers were FITC-CD8 (SK1), APC R700-CD3 (UCHT1), BV510-CD4 (SK3), BV421-IL2 (5344.111), APC-TNF-α (6401.1111), and BV650-IL17A (N49-653), from BD Biosciences, as well as using PerCP-Cy5.5-IFN-γ (4S.B3) from eBioscience-Invitrogen. Flow cytometry was performed on a FACS Celesta flow cytometer with FACS Diva software v.7 (Becton Dickinson, Franklin Lakes, NJ, USA). The lymphocyte gating was set by forward and side scatter and at least ∼100,000 lymphocyte events were acquired. Data were collected and analyzed using Flow Jo 10.7.1 software (TreeStar Inc., Ashland, CA, USA). All data are depicted as frequencies of CD4^+^ and CD8^+^ T cells expressing cytokine(s) and other immune markers.

### Statistical analyses

The data were analyzed statistically with the geometric mean of each group. The differences between the three groups were analyzed with the Kruskal–Wallis test and with Dunn's multiple comparisons test. The comparative analyses between baseline and post-treatment was done by applying the non-parametric Wilcoxon matched-pairs signed rank test. All the above analyses were performed by using GraphPad PRISM Version 9.4. (GraphPad Software, Inc., San Diego, CA, USA). The heat map representation showing hierarchical clusters was done with R by applying the Euclidean distance measure and Ward.d2 method after normalizing the frequencies of cytokine expressions by CD4^+^ and CD8^+^ T cells with the z-score.

## Results

### Enhanced frequencies of SARS-CoV-2-specific CD4^+^ Th1 and Th17 cells in MIS-C

**Figure 1:**
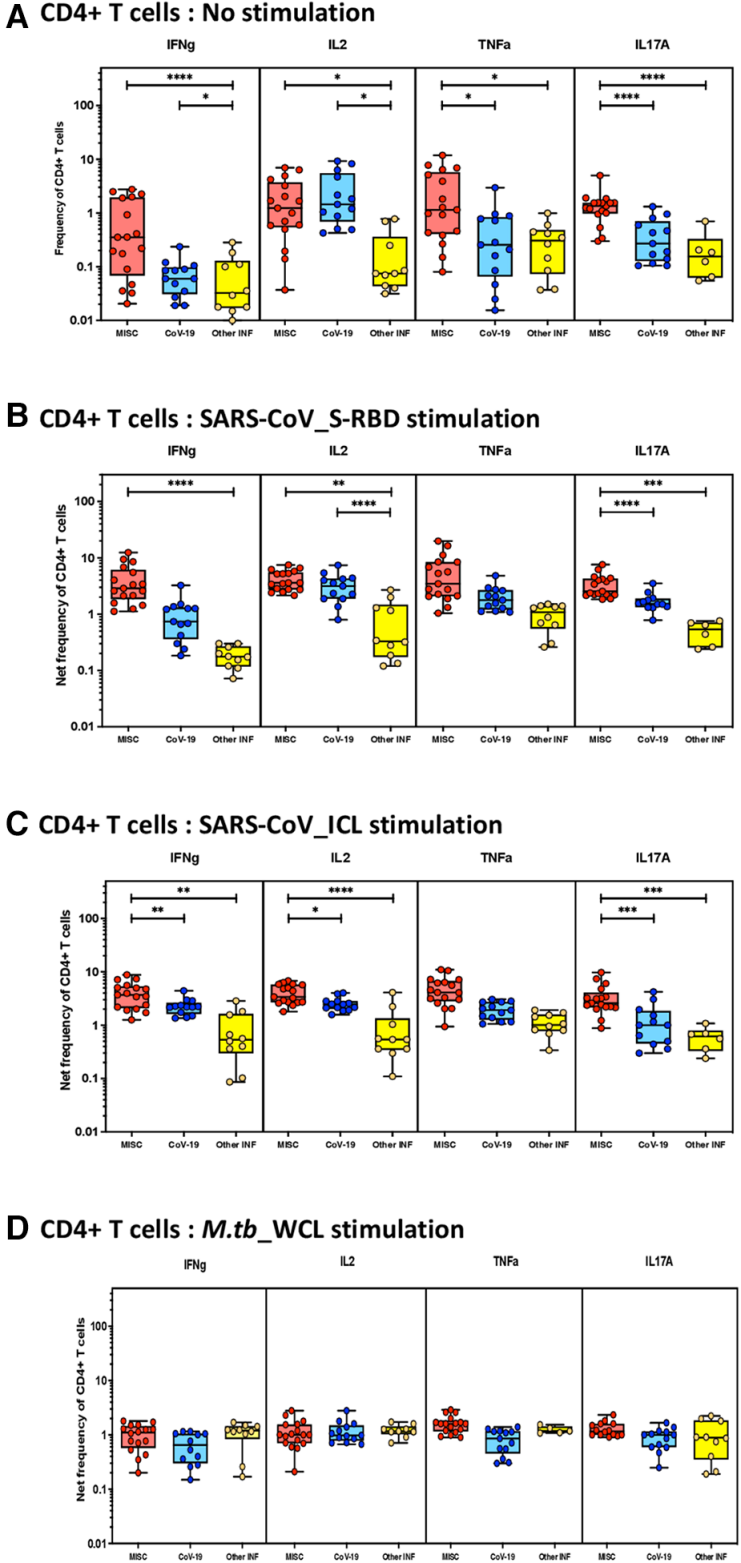
Children with MIS show enhanced frequencies of SARS-CoV-2-specific CD4^+^ Th1 and Th17 cells. The net frequencies of Th1 and Th17 cytokines by CD4^+^ T cells with or without SARS-CoV-2-antigenic stimulation were compared statistically among MIS-C, children with COVID-19, and other infectious diseases. (**A**) CD4^+^ T cells at baseline or no stimulation; (**B**) CD4^+^ T cells at SARS-CoV S-RBD stimulation; (**C**) CD4^+^ T cells at SARS-CoV ICL stimulation; and (**D**) CD4^+^ T cells at *M.tb* WCL stimulation. Each circle in the scatter plot represents a single variable from an individual and the line at the center of the scatter plot is the geometric mean. The *p*-values were calculated using the Kruskal–Wallis test to show the significance and were represented as *, **, ***, and ****.

To elucidate the impact of MIS-C on CD4^+^ T-cell responses, we measured the frequencies of CD4^+^ T cells expressing IFN-γ IL-2, TNF-α, and IL-17A at baseline (no stimulation) and following stimulation with either SARS-CoV-2-specific antigens or *M.tb* WCL. The gating strategy and a representative flow cytometry contour plot are shown in Supplementary Figure S1A. As shown in Figure 1A, at baseline, there were significantly increased frequencies of CD4^+^ T cells expressing IFN-γ IL-2, TNF-α, and IL-17A in MIS-C compared with children with COVID-19 or with other infectious diseases. In response to SARS-CoV-2 S-RBD (Figure 1B) and SARS-CoV-2 ICL (Figure 1C), we observed significantly increased frequencies of CD4^+^ T cells expressing IFN-γ, IL-2, and IL-17A in MIS-C compared to children with COVID-19 and other infectious diseases. In contrast, no significant differences were seen in the frequencies of CD4^+^ T cells expressing cytokines upon *M.tb* WCL stimulation (Figure 1D).

### Enhanced frequencies of SARS-CoV-2-specific CD8^+^ Tc1 and Tc17 cells in MIS-C

**Figure 2:**
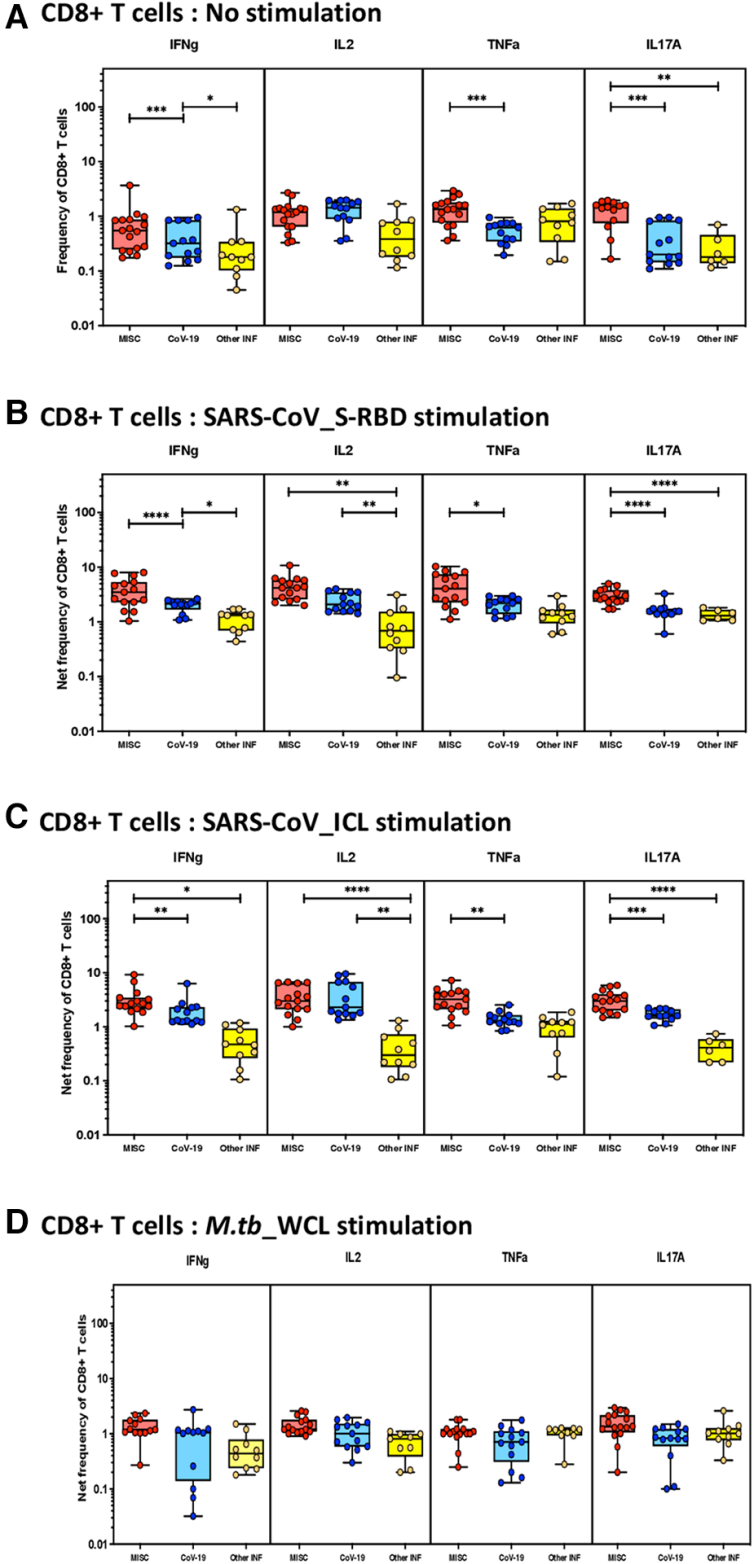
Children with MIS show enhanced frequencies of SARS-CoV-2-specific CD8^+^ Tc1 and Tc17 cells. The net frequencies of Tc1 and Tc17 cytokines by CD8^+^ T cells with or without SARS-CoV-2-antigenic stimulation were compared statistically among children with MIS, COVID-19, and other infectious diseases. (**A**) CD8^+^ T cells at baseline or no stimulation; (**B**) CD8^+^ T cells at SARS-CoV S-RBD stimulation; (**C**) CD8^+^ T cells at SARS-CoV ICL stimulation; and (**D**) CD8^+^ T cells at *M.tb* WCL stimulation. Each circle in the scatter plot represents a single variable from an individual and the line at the center of the scatter plot is the geometric mean. The *p*-values were calculated using the Kruskal–Wallis test to show the significance and were represented as *, **, ***, and ****.

To elucidate the impact of MIS-C on CD8^+^ T-cell responses, we measured the frequencies of CD8^+^ T cells expressing IFN-γ, IL-2, TNF-α, and IL-17A at baseline (no stimulation) and following stimulation with either SARS-CoV-2-specific antigens or *M.tb* WCL. The gating strategy and a representative flow cytometry contour plot are shown in Supplementary Figure S1B. As shown in Figure 2A, at baseline, there were significantly increased frequencies of CD8^+^ T cells expressing IFN-γ, TNF-α, and IL-17A in MIS-C compared to children with COVID-19 and other infectious diseases. In response to SARS-CoV-2 S-RBD (Figure 2B) and SARS-CoV-2 ICL (Figure 2C), we observed significantly increased frequencies of CD8^+^ T cells expressing IFN-γ, IL-2, TNF-α, and IL-17A in MIS-C compared to children with COVID-19 and other infectious diseases. In contrast, no significant differences were seen in the frequencies of CD8^+^ T cells expressing cytokines upon *M.tb* WCL stimulation (Figure 2D). A sub-analysis was conducted to assess the differences in immune responses in the cases of MIS-C among the children aged less than 5 years (*n* = 7) and those greater than 5 years (*n* = 8). We focused on CD4^+^ and CD8^+^ T cells expressing IFN-γ, IL-2, TNF-α, and IL-17A both at baseline (no stimulation) and upon antigen stimulation. The results revealed moderate statistical differences in CD4^+^ T cells for IFN-γ expression under no stimulation conditions and in CD8^+^ T cells for IL-2 expression (no stimulation) and IL-17A expression (RBD stimulation). However, for the rest of the analyzed cytokines, no significant differences were observed among the younger and older children with MIS (see Supplementary Table S1 for detailed data).

### T-cell signatures for MIS-C upon stimulation with SARS-CoV-2 antigen

**Figure 3:**
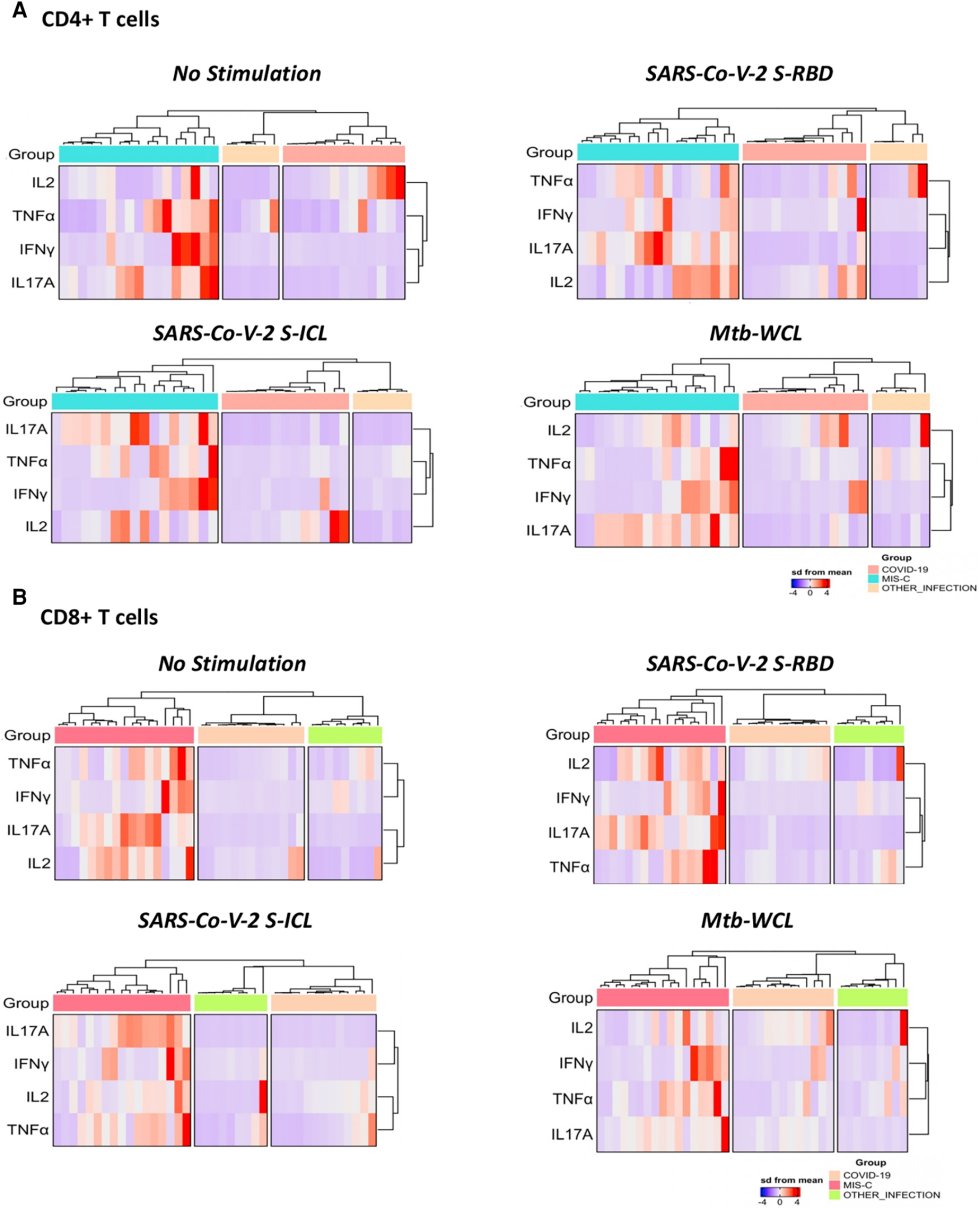
A signature profile of CD4^+^ T. (**A**) Cells expressing Th1/17 cytokines and CD8^+^ T. (**B**) Cells expressing Tc1/17 cytokines upon SARS-CoV-2-specific antigen stimulation in MIS-C, children with COVID-19, and children with other infectious diseases.

To understand the differentiation of T cells upon SARS-CoV-2-specific antigen stimulation, a signature pattern was established for CD4^+^ Th1/17 and CD8^+^ Tc1/17 cytokines. The representative hierarchical cluster shows the distinction of MIS-C from the COVID-19 group and other infections group in both CD4^+^ and CD8^+^ T cells. The Th1/17 and Tc1/17 cytokine expressions were clustered separately between the study groups, and the signatures were seen with no stimulation and with stimulation of SARS-CoV-2 S-RBD and SARS-CoV-2 ICL antigens (Figures 3A,B).

### Antigen-specific CD4^+^ and CD8^+^ T-cell cytokine expressing frequencies are significantly diminished 6–9 months post recovery

**Figure 4:**
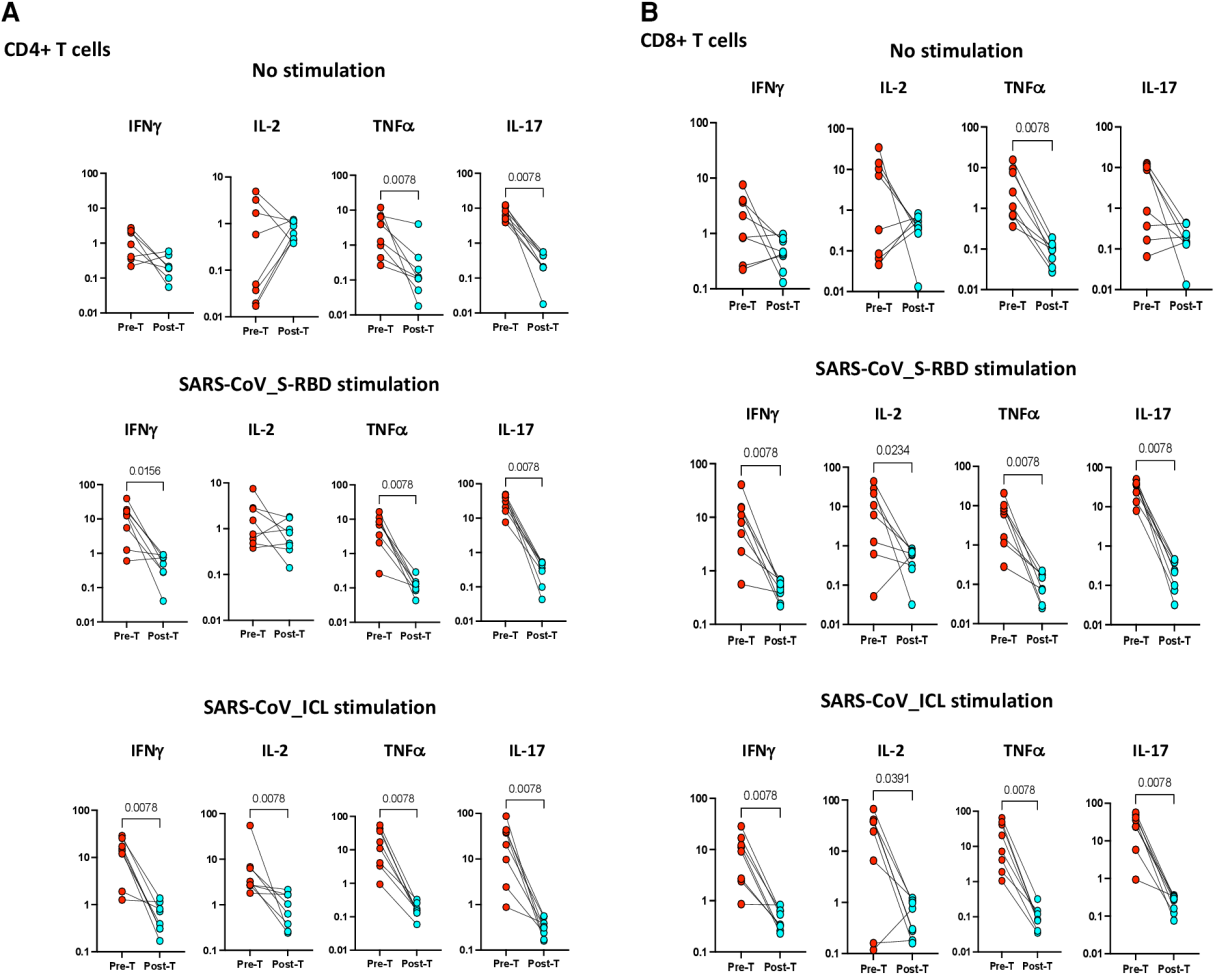
(**A**) Children with MIS show diminished frequencies of SARS-CoV-2-specific CD4^+^ Th1 and Th17 cells after treatment. The net frequencies of Th1 and Th17 cytokines by CD4^+^ T cells with or without SARS-CoV-2 antigenic stimulation were compared statistically between pre- and post-treatment of MIS-C. (Top) CD4^+^ T cells at baseline or no stimulation; (Middle) CD4^+^ T cells at SARS-CoV S-RBD stimulation; and (Bottom) CD4^+^ T cells at SARS-CoV ICL stimulation. Each circle in the scatter plot represents a single variable from an individual and the line connecting to the circle of the same individual from pre- to post-treatment. The *p*-values were calculated using the Wilcoxon test for matched pairs. (**B**) Children with MIS show diminished frequencies of SARS-CoV-2-specific CD8^+^ Tc1 and Tc17 cells after treatment. The net frequencies of Tc1 and Tc17 cytokines by CD8^+^ T cells with or without SARS-CoV-2 antigenic stimulation were compared statistically between pre- and post-treatment of MIS-C. (Top) CD8^+^ T cells at baseline or no stimulation; (Middle) CD8^+^ T cells at SARS-CoV S-RBD stimulation; and (Bottom) CD8^+^ T cells at SARS-CoV ICL stimulation. Each circle in the scatter plot represents a single variable from an individual and the line connecting to the circle of the same individual from pre- to post-treatment. The *p*-values were calculated using Wilcoxon test for matched pairs.

To examine whether the elevated frequencies of CD4^+^ Th1 and Th17 cells are directly linked with MIS-C, we measured the frequencies of these CD4^+^ T cells in MIS-C before and after treatment/recovery (pre vs. post). As shown in Figure 4A, at 6–9 months following recovery, the frequencies of CD4^+^ Th1 and Th17 cells were significantly diminished compared with pre-treatment levels at both baseline (no stimulation) and as well as upon SARS-CoV-2 antigen stimulation. Similarly, as shown in Figure 4B, at 6–9 months following recovery, the frequencies of CD8^+^ Tc1 and Tc17 cells were significantly diminished compared with pre-treatment levels at both baseline (no stimulation) and as well as upon SARS-CoV-2 antigen stimulation.

## Discussion

The severe after-effects of COVID-19 infection sometimes manifest themselves as MIS-C that clinically overlaps with many inflammatory disorders (e.g., Kawasaki disease) (19). Our study focuses on the poorly understood immunologic phenomenon of MIS-C in association with COVID-19. We investigated T-cell responses to stimulation with SARS-CoV-2-specific antigens, since T cells are important for host protection. A modified immune pattern with cytokine storm, lymphopenia, innate and adaptive immune activation, and anti-viral and autoantibody production affecting physiological homeostasis are the characteristics of MIS-C (20). Previous studies have shown that children with MIS have presented with lymphopenia, which would thereby lead to a weakened immune system in the host (2). A cellular study on MIS-C immune signatures showcases reduced CD4^+^ and CD8^+^ T-cell memory subsets in children with MIS (14). Similarly, the downregulation of host-protecting T-cell responses was suggested as evidence of MIS-C and COVID-19 infection (21). Therefore, understanding the immune perturbations after COVID-19 infection may guide in delineating the pathogenesis of MIS-C.

In our study, children with MIS exhibited elevated CD4^+^ and CD8^+^ T cells expressing IFN-γ, IL2, TNF-α, and IL17A. The present study parallels that seen previously in which increased T-cell responses against SARS-CoV-2-specific peptides were observed in most children with MIS (17, 22). Also, the children with MIS have a more significant T-cell response against SARS-CoV-2 antigen than those without MIS (23). An immune profiling study by Vella et al. has shown the trend of lymphopenia with extreme T-cell activation and proliferation in children with MIS (24). The T cells expressing cytokines upon SARS-CoV-2-specific antigens were seen enhanced in MIS-C than in children and adults with COVID-19 infection alone (23). Similarly, another study subsequently reported CD4^+^ Th response and cytotoxic T-cell responses in the MIS-C cohort were elevated in comparison with children with COVID-19 (17). Previous studies from our lab have reported that immuno-phenotyping of the T-cell subsets showed a reduced CD4^+^ and CD8^+^ stem cell memory, central memory, effector memory, and terminal memory in children with MIS and reversal after recovery (14). However, a recent study by Rybkina et al. has shown that there are no statistical differences in the total CD4^+^ and CD8^+^ T-cell responses among acute MIS-C and children with COVID-19 (25).

Previously, our group has shown an increased level of cytokines in the antigen-specific culture supernatants including IFNγ, IL-2, IL-4, IL-13, and IL-17 in MIS-C in comparison with acute COVID-19 and other infectious diseases (16). However, in the current study, we have elucidated the antigen-specific cellular immune responses, and our results revealed that enhanced frequencies of SARS-CoV-2-specific CD4^+^ and CD8^+^ T cells expressing type 1 and type 17 cytokines are major characteristics of the MIS-C immune profile. In addition, in this current study, we compared the SARS-CoV-2-specific T-cell response in MIS-C to individuals presenting with other infections, such as dengue fever, enteric fever, and scrub typhus. The results clearly reveal that the enhanced antigen-specific response we observe in this study are only in MIS-C and children with COVID-19. In addition, we have also validated the specificity of antigenic response with the stimulation of *M.tb*-specific WCL antigen as control.

In most cases, MIS-C requires only symptomatic treatment, but some investigations show treatment of MIS-C with intravenous immunoglobulin (IVIg), corticosteroids, and inflammation inhibitors. Globally, the long-term complications of MIS-C remain unclear (26, 27). We found a pattern of decreased T-cell activation when we observed the SARS-CoV-2-specific antigen stimulation after 6–9 months of treatment in children with MIS. This observation was applicable to both CD4^+^ and CD8^+^ T cells. Our group has previously reported these diminished inflammatory marker responses after MIS-C in cell culture supernatants (16). The mechanism behind the alteration of T-cell activation between the pre- and post-treatment of MIS-C has to be further explored. Also, various other factors were seen that support inflammation during the MIS-C condition (28).

The incidence of T-cell alterations is one of the common features of MIS-C (29). Our strengths include the following: (1) Control children with febrile illness not previously reported in other cohorts (dengue, typhoid fever, scrub typhus); (2) Samples obtained from all children before immunomodulation; and (3) Long-term follow up of children. Our findings reinforce that MIS-C is characterized by enhanced antigen-specific immune responses, which while contributing to host resistance may also enhance host pathology and lead to organ damage. Our study adds to the growing literature in the field that MIS-C is a syndrome of complex immunological etiology. Findings from this study also clearly reveal that enhanced SARS-CoV-2 antigen–specific T-cell responses are an important immunological feature of MIS-C at baseline. Our study suffers from the limitation of a small sample size, and the likely disparity in timing since infection onset with SARS-CoV-2 in the MIS-C and acute COVID-19 groups. Our study also suffers from the limitation of not including children with other autoimmune conditions such as systemic lupus erythematosus and juvenile idiopathic arthiritis. However, conducting the study posed a challenge as these patients were already on immunosuppressive medications, which could potentially interfere with the immune assays we conducted. Nevertheless, our study adds to the growing body of evidence of alterations in T-cell responses, especially cytokine production, which is a key characteristic defect in MIS-C. In conclusion, the findings from the present study advance our knowledge on the immunology of MIS-C and COVID-19 in children, and may benefit researchers.

## Data Availability

The original contributions presented in the study are included in the article/**Supplementary Material**, further inquiries can be directed to the corresponding author.

## Supplementary material

Supplementary Figure S1 Gating strategy for T cells expressing type1 and type17 cytokines upon SARS-CoV-2-specific antigens. The gating strategy shows the whole blood flow analysis from the lymphocyte population. From the CD3^+^ T-cell gating, the CD4^+^ T helper and CD8^+^ T cytotoxic cells were gated. The type-1 and type-17 cytokine-expressing T-cell subsets gated from each T-cell subset are also shown.

Supplementary Table S1 Moderate changes seen between the MIS-C children with less then 5 years (*n*=7) vs greater than 5 years. MIS-C children frequencies and net frequencies of CD4^+^ T cells and CD8^+^ T cell populations expressing Th1 and Th17 cytokines with or without SARS-CoV-2 antigenic stimulation were compared statistically between of less then 5 years (*n*=7) and greater than 5 years (*n*=8). The P-values were calculated using Mann-Whitney test to show the significance and were represented as *p*<0.005.
