# Supplementary figures and images for: Multisystem inflammatory syndrome in children characterized by enhanced antigen-specific T-cell expression of cytokines and its reversal following recovery
Source: Front Pediatr. 2023 Dec 5;11:1235342. doi: 10.3389/fped.2023.1235342 (PMC10728284; doi:10.3389/fped.2023.1235342)

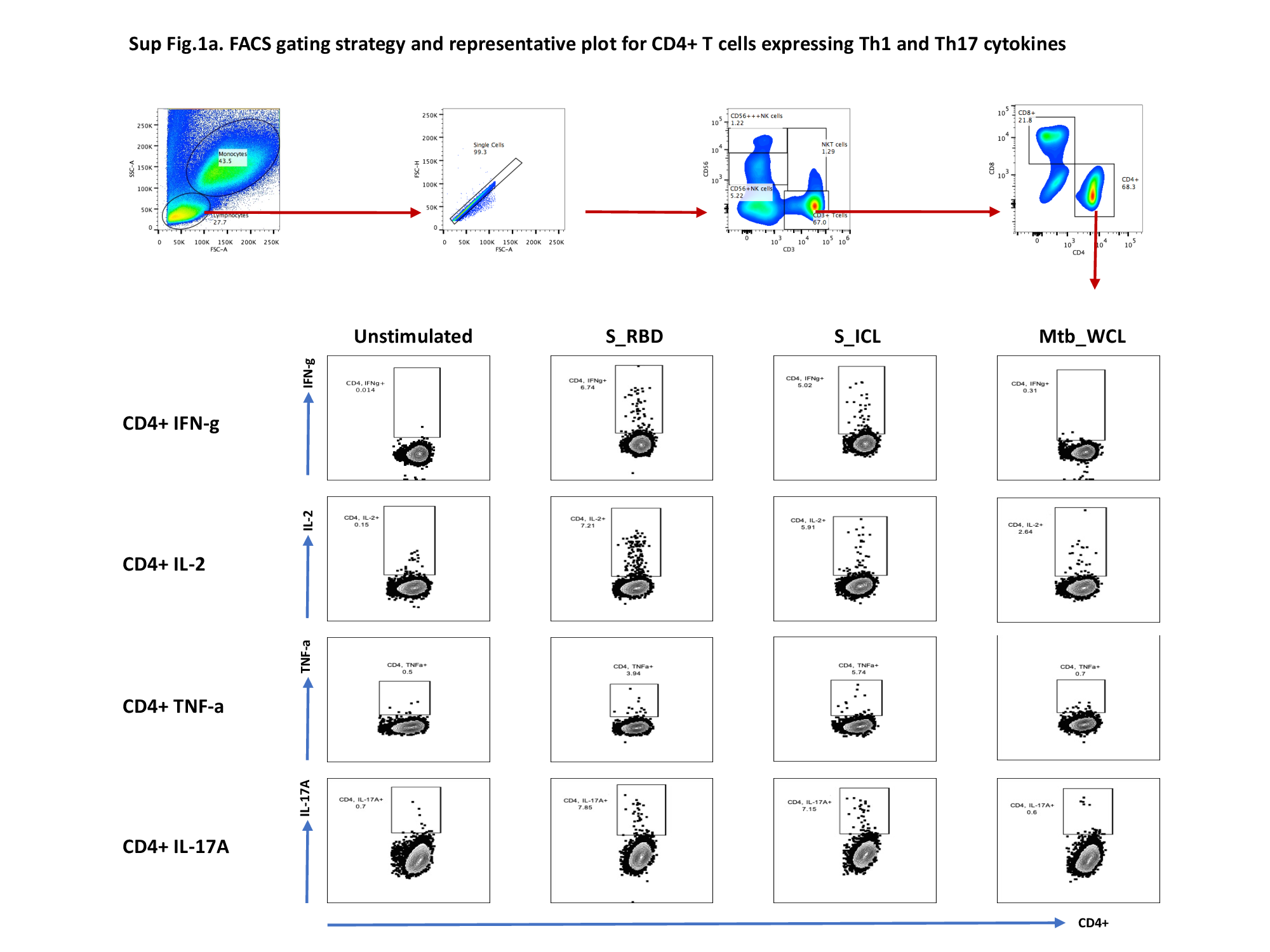

Supplement: Supplementary Figure S1 — Gating strategy for T cells expressing type1 and type17 cytokines upon SARS-CoV-2-specific antigens. The gating strategy shows the whole blood flow analysis from the lymphocyte population. From the CD3+ T-cell gating, the CD4+ T helper and CD8+ T cytotoxic cells were gated. The type-1 and type-17 cytokine-expressing T-cell subsets gated from each T-cell subset are also shown. [file Image1.tiff]
