# Supplementary material for: Multisystem inflammatory syndrome in children characterized by enhanced antigen-specific T-cell expression of cytokines and its reversal following recovery
Source: Front Pediatr. 2023 Dec 5;11:1235342. doi: 10.3389/fped.2023.1235342 (PMC10728284; doi:10.3389/fped.2023.1235342)
Supplement: Supplementary Table S1 — Moderate changes seen between the MIS-C children with less then 5 years (n=7) vs greater than 5 years. MIS-C children frequencies and net frequencies of CD4+ T cells and CD8+ T cell populations expressing Th1 and Th17 cytokines with or without SARS-CoV-2 antigenic stimulation were compared statistically between of less then 5 years (n=7) and greater than 5 years (n=8). The P-values were calculated using Mann-Whitney test to show the significance and were represented as p<0.005. [file Table1.pdf]

Supplementary Table 1 :

CD4+ T cells Responses	UNS			RBD			ICL		
**GeoMean**	**<5 Years**	**>5 Years**	**pvalue**	**<5 Years**	**>5 Years**	**pvalue**	**<5 Years**	**>5 Years**	**pvalue**
IFNγ	0.8789	0.1737	**0.0401**	4.937	2.632	0.1206	3.354	3.712	0.9623
IL-2	0.6673	1.59	0.2359	3.355	4.31	0.327	3.238	4.005	0.3922
TNFα	2.031	0.8171	0.2766	5.09	3.639	0.3638	4.18	3.886	0.536
IL-17A	1.321	1.173	0.9591	4.185	2.521	0.0521	3.314	2.491	0.1738
**CD8+ T cells Responses**	**UNS**			**RBD**			**ICL**		
**GeoMean**	**<5 Years**	**>5 Years**	**pvalue**	**<5 Years**	**>5 Years**	**pvalue**	**<5 Years**	**>5 Years**	**pvalue**
IFNγ	0.7422	0.3839	0.0927	3.906	2.881	0.3969	2.485	3.242	0.0993
IL-2	0.7572	1.311	**0.0339**	4.278	3.835	0.8741	2.872	3.219	0.6943
TNFα	1.055	1.388	0.2652	3.984	3.336	0.7559	3.477	2.613	0.152
IL-17A	0.7731	1.552	0.345	3.563	2.387	**0.0062**	3.606	2.428	0.0939
